# Marrow Fat and Bone: Review of Clinical Findings

**DOI:** 10.3389/fendo.2015.00040

**Published:** 2015-03-30

**Authors:** Ann V. Schwartz

**Affiliations:** ^1^Department of Epidemiology and Biostatistics, University of California San Francisco, San Francisco, CA, USA

**Keywords:** bone marrow fat, osteoporosis, bone marrow fat composition, magnetic resonance spectroscopy, bone marrow lipids

## Abstract

With growing interest in the connection between fat and bone, there has been increased investigation of the relationship with marrow fat in particular. Clinical research has been facilitated by the development of non-invasive methods to measure bone marrow fat content and composition. Studies in different populations using different measurement techniques have established that higher marrow fat is associated with lower bone density and prevalent vertebral fracture. The degree of unsaturation in marrow fat may also affect bone health. Although other fat depots tend to be strongly correlated, marrow fat has a distinct pattern, suggesting separate mechanisms of control. Longitudinal studies are limited, but are crucial to understand the direct and indirect roles of marrow fat as an influence on skeletal health. With greater appreciation of the links between bone and energy metabolism, there has been growing interest in understanding the relationship between marrow fat and bone. It is well established that levels of marrow fat are higher in older adults with osteoporosis, defined by either low bone density or vertebral fracture. However, the reasons for and implications of this association are not clear. This review focuses on clinical studies of marrow fat and its relationship to bone.

## Marrow Fat in Humans

Marrow fat is distinct from white adipose tissue and from brown adipose tissue (1, 2). The marrow fat depot also appears to be under separate control from other fat depots. Humans have virtually no marrow fat at birth, but marrow adiposity increases with age, particularly during the third decade of life. This well-known conversion of “red” to “yellow” marrow occurs to varying degrees at different skeletal sites, with a high percentage in the axial skeleton and a lower percentage in the vertebral bodies. For example, Justesen et al. reported that marrow fat in post-mortem iliac crest bone biopsies increased from 40% at age 30 to 68% by age 100 years (3). Both the size and number of adipocytes increase with age (4). The lifetime pattern of vertebral BMF accumulation appears to differ in men and women (5, 6). Griffith et al. found that before age 55 years, BMF is higher in men, but from ages 55–65 years, women experience a steeper increase in BMF while men have a gradual increase in BMF with aging (5). As a result, BMF is higher in women at older ages compared with men.

Higher levels of marrow fat are known to occur in humans in conditions of starvation (7, 8), alcoholism (9), spinal cord injury (10), and prolonged bed rest (11), conditions that are also associated with reduced bone density. Medications that increase marrow fat include glucocorticoids (12) and thiazolidinediones (TZDs) (13), highlighting the interconnection of bone formation and marrow fat accumulation.

The age-related changes in marrow fat and the increases with specific pathologies have been recognized for years, but progress in understanding its role and functions has been relatively recent. *In vitro* and animal models have provided new insight into the connection between marrow fat and bone in particular, and these findings have been extensively reviewed (14–17). Briefly, studies indicate that marrow fat may function as an indirect marker of changes in bone and may also play a direct role in bone health. Changes in the differentiation programs that give rise to osteoblasts and adipocytes from mesenchymal stem cells in the marrow can favor adipogenesis over osteoblastogenesis, leading to increased marrow fat with reduced bone formation. Major control of mesenchymal stem cell differentiation resides with peroxisome proliferator-activated receptor (PPAR)-γ for adipogenesis and runt-related transcription factor 2 (RUNX2) for osteogenesis, with the canonical Wnt/β-catenin pathway playing a central role. As with other fat depots, marrow fat is an endocrine organ and may directly influence the survival and function of osteoblasts and osteoclasts through the release of cytokines, adipokines, and fatty acids.

## Non-Invasive Measurement of Marrow Fat Propels Clinical Research

Progress in clinical studies of marrow fat and bone has been greatly facilitated by recent technical advances in our ability to non-invasively measure marrow fat. Historically, clinical measures of marrow fat required a biopsy. More recently, non-invasive measurements have become available in the research setting although these are not generally used clinically. Two techniques using magnetic resonance imaging (MRI) are widely used to assess fat in the bone marrow: magnetic resonance spectroscopy (MRS) and T1-weighted MRI. Dual energy QCT can also be used to quantify marrow fat. However, dual energy scans require relatively high levels of radiation. Single energy QCT does not give a reliable measure of marrow fat.

**Figure 1:**
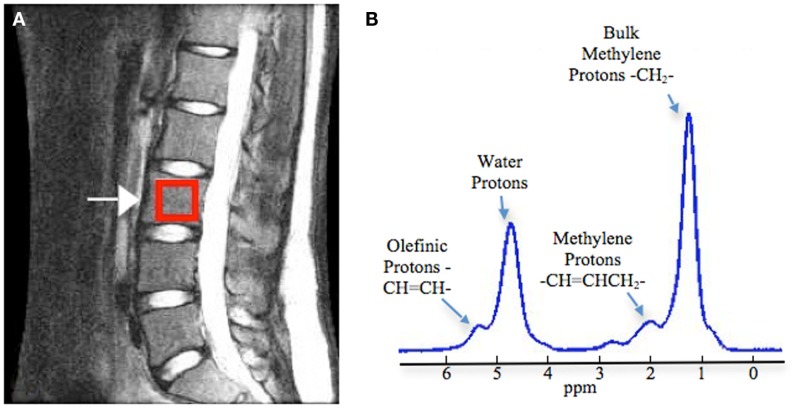
**Measurement of vertebral bone marrow fat using magnetic resonance spectroscopy**. **(A)** Positioning of box in the middle of the vertebral body (L3 as example). **(B)** Example spectrum in vertebral bone marrow (3 T). Four peaks were well resolved: olefinic, double bond –CH =CH– protons at 5.31 ppm, water protons at 4.65 ppm, the CH_2_ methylene protons α to a double bond (–CH =CHCH_2_–), at 2.03 ppm, and the bulk CH_2_ methylene protons at 1.3 ppm. Adapted with permission from Ref. (18).

In MRS measurements, marrow fat is expressed as a percentage rather than an absolute value. For evaluation of vertebral marrow fat, the measurement is acquired on one or more of the lumbar vertebral bodies (L1–L4). A single voxel is placed in the center of the vertebral body (Figure 1) (18). The calculation of marrow fat varies slightly across studies. The percent marrow fat can be calculated using the large lipid peak at 1.3 ppm (saturated lipids), ignoring the much smaller lipid peaks at 5.3 (unsaturated lipids) and 2.0 (residual lipids) (18, 19). Fat content is then calculated as: fat content = [(*I*_fat_/(*I*_fat_ + *I*_water_)] × 100%. With higher resolution MRI (3 T), fat content has also been reported using three of these lipid peaks (20). Fat content is then calculated as: fat content = [(*I*_UL_ + *I*_RL_ + *I*_SL_)/(*I*_UL_ + *I*_RL_ + *I*_SL_ + *I*_water_)] × 100%.

Some studies have relied on one vertebral level while others have reported an average of several levels. The fat content tends to increase from L1 to L4 (18, 21). Thus, reported marrow fat levels may vary across studies depending on which levels were included. In postmenopausal women, vertebral marrow fat levels at separate vertebral levels from L1 to L4 were highly correlated (18). Although trabecular spine vBMD is measured at L1 and L2, the correlation between BMF and trabecular spine vBMD in older women did not vary substantially when each vertebral level was considered separately. The correlations with trabecular spine BMD (L1–L2) were: −0.23 for BMF measured at L1, −0.20 for L2, −0.23 for L3, −0.28 at L4, and −0.26 for the mean L1–L4 (21).

BMF measured by T1-weighted MRI is also expressed as a percentage. Fat fraction is calculated for the whole body and for specific regions, often the pelvic region, from a whole-body MRI (22). In contrast to MRS, the T1-weighted MRI is semi-quantitative and may also have errors due to partial volume effects. However, MRS is limited to measurement of a small area of the total marrow. Shen et al. compared measurements of BMF using MRS, T1-weighted MRI, and a third MR-based method, the Dixon method, and found good correlations among the different methods (23).

With MRS, it is also possible to quantify the smaller lipid peaks and assess the degree of saturation of the lipids in marrow (Figure 1). Yeung et al. introduced the unsaturation index, using spectra acquired on a 1.5 T scanner (24). The signal amplitude at about 5.3 ppm (olefinic) was compared to combined signal amplitudes at 5.3, 2.0, 1.3, and 0.9 ppm, to obtain an unsaturation index. Degree of lipid unsaturation has also been assessed using spectra acquired with a 3 T scanner, with a formula that compares signal amplitude at 5.3 ppm to the combined amplitudes at 5.3 ppm (UL), 2.0 ppm (RL), and 1.3 ppm (SL), UL (%) = [*I*_UL/_(*I*_UL_ + *I*_RL_ + *I*_SL_)] × 100% (20). Resolution of the smaller peaks is difficult, even at 3 T, and the unsaturation index should be utilized as a relative index for comparing lipid composition rather than an absolute measure of marrow lipid unsaturation.

## An Inverse Relationship between Marrow Fat and Bone Density

Cross-sectional studies in humans using different methods to assess bone density and marrow fat have found that lower bone density is accompanied by higher marrow fat. In 1971, Meunier et al. reported that samples from the iliac crest in women with osteoporosis had a pronounced accumulation of adipocytes, relative to age-matched controls (25). Other studies using bone biopsy to assess marrow fat have also reported a relationship with osteoporosis (3, 25, 26). Histomorphometry has shown a negative association between bone marrow fat and bone formation rates (26).

Studies using non-invasive imaging methods to assess BMF have also reported an association between higher BMF and lower BMD (18, 19, 21, 27, 28). Most studies of BMF and bone density have relied on DXA measurements of bone. Negative associations between BMF and BMD by DXA have been reported for men (19) and women (27). Although bone density by DXA is a robust predictor of fracture risk, the DXA measurements provide only areal estimates of density, cannot distinguish cortical and trabecular bone, and are thus limited for purposes of etiologic research. QCT measurements of bone can potentially provide greater insight into the aspects of bone metabolism that are associated with BMF. In a study of postmenopausal women with and without type 2 diabetes, trabecular spine vBMD and BMF were negatively correlated in both groups (29). In the Iceland AGES–Reykjavik cohort of older adults, higher BMF was associated with lower trabecular spine, total hip and femoral neck vBMD, and reduced vertebral compressive strength, derived from QCT, in women (21). A difference of about +8% in BMF was associated with differences in trabecular vBMD of −10.5% (95% CI: −17.2 to −3.2%) at the spine, −7.08% (−12.51 to −1.31) at femoral neck, and −3.95% (−7.64 to −0.12%) at total hip. In men, BMF was also negatively associated with trabecular spine vBMD and with vertebral compressive strength, but associations were not statistically significant. In men, there was no evident association between BMF and trabecular vBMD at the hip. No associations were seen with cortical vBMD and BMF at either hip site in men or women.

These cross-sectional studies indicate that higher BMF is associated with reduced bone density and strength, but longitudinal data on these relationships are limited. Importantly, Griffith et al. reported that higher BMF at the hip predicts femoral neck bone loss, measured by DXA, over 4 years in postmenopausal women (mean age 74 years) (30). Women with BMF above and below the median experienced average bone loss of 1.6 and 4.7%, respectively. Changes in BMF were not measured in this study.

DXA and QCT measurements of bone are both vulnerable to artificially low readings in the presence of higher BMF (31, 32). Blake et al. estimated that BMF and BMD by DXA are still negatively correlated even after the artificial lowering of BMD with higher BMF is taken into account (32). It is also reassuring that studies using biopsy methods rather than imaging to assess bone and marrow fat have reported negative associations (3, 25, 26).

## Marrow Fat and Fracture

Studies have reported a relationship between higher BMF and prevalent vertebral fracture (3, 21, 33, 34). Justesen et al. reported higher marrow fat, measured in iliac crest biopsies, in 26 women with prevalent vertebral fracture compared with age-matched controls (63 vs. 54%, *p* = 0.02) (3). Wehrli et al. also found higher marrow fat in those with prevalent vertebral deformities in a study of older adults (106 women, 33 men) (33). Mean BMF at the spine was 55 and 45% (*p* < 0.001) in those with and without a baseline vertebral fracture, respectively, and remained statistically different after adjustment for spine BMD by DXA. In AGES–Reykjavik, higher vertebral BMF was associated with prevalent vertebral fracture (56.9 vs. 53.7%, *p* = 0.01), in models adjusted for age, gender, and trabecular spine vBMD (21). A history of non-vertebral fracture was not associated with BMF in postmenopausal women (20) or in the Iceland AGES cohort (21). To date, studies of BMF and incident fracture are not available.

## Marrow Fat and Sclerostin

Sclerostin is a product of osteocytes that is a negative regulator of bone formation and the target of new therapies for osteoporosis. Some (35, 36) but not other (37, 38), longitudinal studies have reported an increased risk of incident fracture with higher sclerostin. Somewhat surprisingly, higher circulating sclerostin levels are associated with higher bone density (35, 37–40). Among older adults in the Iceland AGES–Reykjavik cohort, serum sclerostin was positively associated with vertebral bone marrow fat in men, but not in women (41). These results indicate a possible relationship between osteocyte activity and bone marrow fat.

## Possible Gender Differences in the Relationship between Marrow Fat and Bone

Cross-sectional results from the Iceland AGES–Reykjavik cohort suggest that the relationships between BMF, bone density, and bone strength, as well as sclerostin, may differ by gender. Stronger correlations were observed between BMF and bone in women, but BMF was associated with prevalent vertebral fracture in men (21). These gender differences may be due to the effects of sex hormone levels on BMF and bone. Estrogen as well as testosterone levels are higher in older men compared with postmenopausal women. There is evidence from animal and clinical studies that sex hormone levels affect BMF. Rodents experience increased marrow adiposity with ovariectomy along with decreased bone mass (42), and conversely estrogen replacement reduces bone marrow adipogenesis in older mice (43). Female rats receiving testosterone have also been shown to have lower BMF content (44). In postmenopausal women, treatment with transdermal estrogen reduced BMF measured in bone biopsies (45). Studies of the relationship between endogenous estrogen or testosterone levels and BMF in older adults are not currently available.

## Marrow Fat and Cortical Porosity

Investigation of the relationship between marrow fat and bone has focused on the amount and composition of fat in the marrow cavity. With the growing appreciation of cortical porosity as a distinct factor in bone health and strength, there is now also interest in assessing the presence of marrow fat within cortical pores. New techniques using high-resolution peripheral computed quantitative tomography (HRpQCT) and 3 T MRS show promise in distinguishing marrow and blood vessels in pore space (46). Future studies that can identify the extent of marrow fat in cortical pores may help to identify underlying mechanisms determining porosity.

## Lipid Composition of Marrow Fat also Associated with Bone Density and Fracture

The composition as well as the quantity of marrow fat may be relevant for skeletal health (47). The relative amounts of saturated and unsaturated lipids in marrow fat can be measured non-invasively with MR spectroscopy, allowing exploration of this aspect of marrow fat composition. Small studies suggest that a lower proportion of unsaturated lipids in vertebral marrow fat may be associated with reduced BMD and greater prevalence of fracture (20, 24). Yeung et al. assessed vertebral marrow fat content and composition in 12 premenopausal controls and 50 older women (60+) without evidence of vertebral fracture (24). The older women included those with normal (*n* = 15), low (*n* = 15), and osteoporotic (*n* = 20) spine BMD. Consistent with other reports, marrow fat content was 29% in young control women, and 56, 63, and 65% in older women with normal, low, and osteoporotic BMD, respectively. The highest proportion of unsaturated lipids in marrow fat was found in the younger controls (0.127) and the lowest proportion in older women with osteoporotic spine BMD (0.091). Unsaturation levels in older women with normal and osteopenic BMD were 0.114 and 0.097, respectively. The unsaturation level and marrow fat content were negatively correlated (*r* = −0.53, *p* < 0.0001). In a study of postmenopausal women (*n* = 69), Patsch et al. reported an association between history of fracture and lower unsaturation levels, even after adjustment for spine vBMD (−1.7%; 95% CI −2.8 to −0.5%) (20).

Marrow fat specimens obtained by iliac crest aspiration during hip surgery in 24 postmenopausal women were analyzed with high-resolution (11.7 T) MR (48). Similar to above studies using non-invasive measurements, the unsaturation level was lower in those with low BMD compared to those with normal BMD. However, in another study using marrow fat specimens obtained during elective orthopedic surgery, gas chromatography analysis of fatty acids found similar composition in those with normal, low, and osteoporotic BMD in all specimens (*n* = 126), in those from the tibia (*n* = 80), and in those from the proximal femur (*n* = 32) (2).

## Marrow Fat, Other Fat Depots, and Diabetes

The role of marrow fat, including the underlying mechanisms of its relationship to bone, remains poorly understood. One important area of clinical investigation has considered the relationship between marrow fat and other fat depots. The sizes of adipose depots outside the marrow, including total body, visceral, and subcutaneous depots tend to be strongly correlated with each other and with body size. In contrast, BMF tends to be independent of overall body size (3, 22, 49–51) and total body fat (22, 51). However, a study in the CARDIA cohort (*n* = 210) found a negative correlation between BMI and hip BMAT (−0.38; 95% CI −0.53 to −0.21) (52) and a large study (*n* = 455) of men and women aged 18–88 years identified a positive correlation between total body fat and pelvic BMAT (*r* = 0.24, *p* < 0.01) (53). In contrast, marrow fat appears to be consistently elevated in patients with anorexia nervosa who have very low levels of total body fat (7).

Evidence regarding the relationship between visceral adipose tissue and marrow fat is inconsistent. Positive correlations between BMF and visceral adipose tissue (*r* = 0.34, *p* = 0.02) have been reported in obese premenopausal women (50) and in the CARDIA cohort of middle-aged adults for pelvic BMAT (*r* = 0.17, *p* < 0.05) but not hip BMAT (*r* = 0.14, *p* > 0.05) (52). However, other studies have not found an association (22, 29, 49). In a large cross-sectional study (*n* = 455), there was a modest but statistically significant negative correlation between VAT and pelvic BMAT (*r* = −0.10, *p* < 0.001) (53).

Marrow fat is higher with diabetes in rodent models of type 1 diabetes (54), but there have been few clinical studies of this relationship and results are inconsistent. In a study of 16 adult men and women with type 1 diabetes (T1D) and 12 healthy controls, vertebral BMF did not differ by diabetes status (55). In postmenopausal women with (*n* = 13) and without (*n* = 13) type 2 diabetes (T2D), those with T2D had higher BMF (69.3 vs. 67.5%, *p* = 0.31), but the difference was not statistically significant (29). However, in the women with T2D, BMF was positively correlated with A1C (*r* = 0.82, *p* < 0.05). A study in 156 older men reported higher BMF in those with T2D (59 vs. 55%, *p* = 0.03) (56). Marrow fat composition may also differ by diabetes status. Patsch et al. found that T2D was associated with lower unsaturation levels of vertebral marrow fat (−1.3%; 95% CI −2.3 to −0.2%) in postmenopausal women (20).

## Weight Loss, Bone Loss, and Marrow Fat

Weight loss in older adults is known to increase bone loss and fracture risk, even among those who are overweight or obese (57, 58). In states of extreme under-nutrition, BMF is increased while other fat depots are depleted (7), but little is currently known about the clinical effects of weight loss on BMF in overweight or obese adults.

In a short-term (13-week) study of diet-induced weight loss in 55 women and 17 men, age 19–46 years, participants lost an average of 9.2 kg (about 9% of body weight) (59). Pelvic BMAT, measured by whole-body MRI, also decreased by an average of 17.8 cm^3^ (about 8%). However, total body BMD increased by 0.01 g/cm^2^ (about 1%); the lack of bone loss in spite of substantial weight loss may be due to the younger age of participants, use of whole body BMD rather than hip BMD, and/or the short follow-up time. In a 12-week weight loss study (*n* = 10), participants lost 5.2 kg on average, but did not experience a statistically significant change in pelvic or total BMF, measured by MRI (60). In a study of the effects of bariatric surgery, changes in vertebral BMF were assessed before and 6 months after surgery in 11 morbidly obese women who lost on average 36.5% of total body fat. Schafer et al. reported little change in vertebral BMF (+0.9%; 95% CI −10.0 to +11.7%, *p* = 0.84) in women without diabetes (*n* = 5) and an apparent decrease in BMF (−7.5%; 95% CI −15.2 to +0.1%, *p* = 0.05) in those with T2D (*n* = 6) (61). Bariatric surgery can dramatically improve insulin sensitivity (62) and the decrease in BMF in the T2D women may reflect a response to changes in diabetes status. Women did lose bone after the surgery at the total hip (4.1%), femoral neck (5.2%), and spine (7.4%). However, this bone loss, induced by the substantial weight loss of bariatric surgery, was not accompanied by increases in BMF.

These findings suggest that changes in bone and in marrow fat are not necessarily linked and that their inter-relationship is dependent on other factors including diabetes status. Further investigation of the dynamic relationships among weight loss, insulin resistance, bone loss, and changes in marrow fat promises to give us a better understanding of the interplay of marrow fat and bone.

## Summary

Osteoporosis is an important health issue, contributing substantially to morbidity and mortality among older adults. Health care costs for osteoporosis-related fractures in the United States were estimated as $19 billion in 2005 and are expected to increase 50% by 2025 with the growth of the older population (63). Animal studies have identified BMF as part of the dynamic processes influencing bone density and strength, and cross-sectional clinical studies have confirmed relationships between higher BMF, lower bone density, and increased prevalence of vertebral fracture in older men and women while suggesting gender differences in the strength of these relationships. This accumulating evidence points to the potential for BMF to play a diagnostic and therapeutic role in the prevention of osteoporosis (64, 65). At this juncture, longitudinal data are needed to determine the relationship between changes in BMF and bone, particularly in the context of weight loss and changes in other fat depots, and to disentangle the gender differences observed in cross-sectional studies. A better understanding of the role of BMF in humans may lead to new avenues to promote bone formation and thus prevent and treat osteoporosis.

## Conflict of Interest Statement

The author declares that the research was conducted in the absence of any commercial or financial relationships that could be construed as a potential conflict of interest.
